# Supplementary material for: FERN – a Java framework for stochastic simulation and evaluation of reaction networks
Source: BMC Bioinformatics. 2008 Aug 29;9:356. doi: 10.1186/1471-2105-9-356 (PMC2553347; doi:10.1186/1471-2105-9-356)
Supplement: Additional file 1 — FERN distribution, Version 1.3. This archive contains the FERN source code and binaries as well as documentation and example models in FernML and SBML. [file 1471-2105-9-356-S1.zip › fern/doc/javadoc/index-files/index-20.html]

V-Index


---


|  |  |  |
| --- | --- | --- |
| **Overview** \| Package \| Class \| Use \| **Tree** \| **Deprecated** \| **Index** \| **Help** | |  |
| **PREV LETTER**   NEXT LETTER | **FRAMES**    **NO FRAMES**     **All Classes** |  |


A B C D E F G H I J L M N O P R S T U V 

---


## **V**

**Value** - Variable in class fern.network.sbml.MathTree.ConstLeaf: **Value** - Variable in class fern.network.sbml.MathTree.GlobalLeaf: **valueOf(String)** - Static method in enum fern.analysis.NetworkSearchAction.NeighborType: Returns the enum constant of this type with the specified name. **valueOf(String)** - Static method in enum fern.cytoscape.ColorCalculator.Scale: Returns the enum constant of this type with the specified name. **valueOf(String)** - Static method in enum fern.simulation.Simulator.FireType: Returns the enum constant of this type with the specified name. **values()** - Static method in enum fern.analysis.NetworkSearchAction.NeighborType: Returns an array containing the constants of this enum type, in the order they are declared. **values()** - Static method in enum fern.cytoscape.ColorCalculator.Scale: Returns an array containing the constants of this enum type, in the order they are declared. **values()** - Static method in enum fern.simulation.Simulator.FireType: Returns an array containing the constants of this enum type, in the order they are declared. **verbose** - Variable in class fern.simulation.algorithm.AbstractBaseTauLeaping

---


|  |  |  |
| --- | --- | --- |
| **Overview** \| Package \| Class \| Use \| **Tree** \| **Deprecated** \| **Index** \| **Help** | |  |
| **PREV LETTER**   NEXT LETTER | **FRAMES**    **NO FRAMES**     **All Classes** |  |


A B C D E F G H I J L M N O P R S T U V 

---
